# Genome Sequence of *Chlamydia psittaci* Strain 01DC12 Originating from Swine

**DOI:** 10.1128/genomeA.00078-12

**Published:** 2013-01-15

**Authors:** Helena M. B. Seth-Smith, Michelle Sait, Konrad Sachse, Wolfgang Gaede, David Longbottom, Nicholas R. Thomson

**Affiliations:** a Pathogen Genomics, Wellcome Trust Sanger Institute, Wellcome Trust Genome Campus, Hinxton, Cambridgeshire, United Kingdom; b Moredun Research Institute, Pentlands Science Park, Bush Loan, Edinburgh, Midlothian, United Kingdom; c Friedrich-Loeffler-Institute (Federal Research Institute for Animal Health), Institute of Molecular Pathogenesis, Jena, Germany; d State Institute for Consumer Protection of Saxony-Anhalt, Dept. for Veterinary Medicine, Stendal, Germany

## Abstract

*Chlamydia psittaci* is the etiological agent of psittacosis and is a zoonotic pathogen infecting birds and a variety of mammalian hosts. Here we report the genome sequence of the porcine strain 01DC12 which is representative of a novel clade of *C. psittaci* belonging to *ompA* genotype E.

## GENOME ANNOUNCEMENT

*Chlamydia psittaci* is a Gram-negative, obligate intracellular bacterium and is the etiological agent of psittacosis, causing disease primarily in birds but also infecting a variety of mammals, including humans. *C. psittaci* has been isolated from the lungs (1), genital tract (2), and semen (3) of pigs. At least 15 different genotypes have been defined on the basis of the *ompA* gene sequence variation (4). Here we report the genome sequence of 01DC12, a strain that was isolated from a pig in Saxony-Anhalt, Germany.

The *C. psittaci* 01DC12 genome was sequenced using the Illumina HiSeq platform with 75-bp paired-end reads, resulting in a mean genome coverage of 248×. Reads were assembled using Velvet v1.0.12 (5) to produce 8 contigs, which were ordered against the *C. psittaci* strain RD1 genome (6). These were finished manually and using GapFiller (7), resulting in an improved high-quality draft genome sequence (8) consisting of 2 contigs. The chromosome of *C. psittaci* 01DC12 comprises 1,171,011 bp, with G+C content of 39.0%. Annotation was transferred from strain RD1 using annotations_update (https://github.com/sanger-pathogens/annotations_update) on the basis of BLASTN similarity using the default settings, and manually curated using Artemis (9). Comparative analysis with closely related *C. psittaci* strains (10–12) was performed using ACT (13). The remaining gap, estimated at 2599 bp, is predicted to encode C and N termini of repetitive *pmp* genes. This region plus the gaps filled using GapFiller are marked in the genome annotation.

The genome contains 963 predicted coding sequences (CDSs), a single rRNA operon, and 38 tRNA genes. Analysis of the *ompA* gene confirms that *C. psittaci* 01DC12 belongs to genotype E. Sequence analysis of the 7 loci used for multilocus sequence typing (MLST) (14) assigned *gatA*, *hemN*, and *hflX* to allele 11, *enoA* and *fumC* to allele 13, and *gidA* and *oppA* to allele 14, indicating that *C. psittaci* 01DC12 is a novel MLST sequence type. Comparison with the genome of type strain 6BC (10, 11) shows that the 01DC12 chromosome contains deletions of 360 bp and 144 bp, respectively, in two putative membrane proteins (BN356_2661 and BN356_4221) and a deletion of an 802-bp repeat at the C terminus of an *incA*/TMH-family membrane protein (BN356_7881). Comparative mapping against strain 6BC revealed 6,262 single-nucleotide polymorphisms (SNPs), suggesting that strain 01DC12 represents a new lineage within *C. psittaci*. A total of 11 pseudogenes were identified in the genome of 01DC12, including those predicted to encode an aldolase (BN356_2651), Pmp8G (BN356_2811), 6 membrane proteins (BN356_3711, BN356_5531, BN356_5541, BN356_5601, BN356_5741, and BN356_6271), a *Mycobacterium avium* complex (MAC)/perforin-domain protein (BN356_5611), and an IncA-family protein (BN356_7871). The genome of strain 01DC12 includes a single plasmid of 7553 bp with G+C content of 32.8%, predicted to encode 8 CDSs. Plasmid p01DC12 is differentiated from the plasmid carried by strain 6BC by 2 SNPs (10, 11): one synonymous SNP in a putative helicase (BN356_p003) and one nonsynonymous SNP resulting in a T-I change in hypothetical protein BN356_p008.

### Nucleotide sequence accession numbers. 

The genome and plasmid sequences of *C. psittaci* 01DC12 have been deposited in EMBL under accession numbers HF545614 and HF545615.
